# Comparison of Survey Methods in Norovirus Outbreak Investigation, Oregon, USA, 2009

**DOI:** 10.3201/eid1611.100561

**Published:** 2010-11

**Authors:** John Y. Oh, June E. Bancroft, Margaret C. Cunningham, William E. Keene, Sheryl B. Lyss, Paul R. Cieslak, Katrina Hedberg

**Affiliations:** Author affiliations: Centers for Disease Control and Prevention, Atlanta, Georgia, USA (J.Y. Oh, S.B. Lyss);; Oregon Public Health Division, Portland, Oregon, USA (J.Y. Oh, J.E. Bancroft, M.C. Cunningham, W.E. Keene, P.R. Cieslak, K. Hedberg)

**Keywords:** Viruses, enteric infections, food-borne infections, gastroenteritis, norovirus, Internet, questionnaires, epidemiology, survey methods, dispatch

## Abstract

We compared data from an Internet-based survey and a telephone-based survey during a 2009 norovirus outbreak in Oregon. Survey initiation, timeliness of response, and attack rates were comparable, but participants were less likely to complete Internet questions. Internet-based surveys permit efficient data collection but should be designed to maximize complete responses.

Internet-based questionnaires are increasingly used during investigations of outbreaks; however, compared with telephone interviews, a differential response rate on the basis of exposures or outcomes might bias results (*1*–*6*). On September 24, 2009, the Oregon Public Health Division was notified of an outbreak of gastroenteritis that occurred among participants of a 475-mile bicycle ride during September 13–19, 2009. Five of 6 riders who independently reported illness to the event organizer and provided stool specimens were positive for norovirus (GII) infection. In responding to the outbreak, we administered a questionnaire using Internet- and telephone-based methods to directly compare data with regard to response rates, attack rates, and risk factors for illness.

## The Study

The event organizer provided telephone numbers, email addresses, and age and sex information for all 2,273 registered riders, of whom 1,288 were Oregon residents. Separate samples of Oregon cyclists were randomized to participate in identically worded surveys, either over the Internet (n = 204) or by telephone (n = 93). The survey contained 95 questions, including 46 about food items eaten. Survey completion was defined as provision of an answer to the last question in the survey (did the participant become ill?), unless the respondent answered “yes.” An affirmative answer led to additional questions about symptoms of illness. Each survey took ≈10–15 minutes to complete.

The Internet survey was formatted with Inquisite Survey (Inquisite, Inc., Austin, TX, USA). We sent an email message that included a link to the survey to the riders. Among 204 riders selected for the Internet survey, 201 had valid email addresses. A reminder was emailed to nonresponders after 5 days. Of the 93 riders selected for the telephone survey, 91 had valid telephone numbers. Oregon Public Health Division interviewers attempted at least 5 times to telephone each participant, including during the evening.

We defined a case as vomiting or >3 loose stools within 24 hours in an event rider with onset during September 11–22, 2009 (i.e., a period that included the 2 days before and the 3 days after the ride). Analyses were conducted in SAS 9.1 (SAS Institute, Inc., Cary, NC, USA). Statistical tests were performed by using χ^2^ tests with significance determined as p<0.05.

Although similar proportions of participants initiated each survey type (153/201 [76%] Internet vs. 76/91 [84%] telephone) (Table 1), participants in the Internet survey were less likely to complete the survey (129/201 [64%] vs. 72/91 [79%]; p = 0.01 for difference in overall completion rate). Within each subgroup, participants were less likely to complete the Internet survey than the telephone survey (Table 2), although the differences were not significant in each subgroup. Within the Internet survey cohort, riders >50 years of age were more likely to complete the survey (80/114 [70%]) than were riders <50 years (48/86 [56%]; p<0.05).

**Table 1:** Comparison of Internet- and telephone-based survey responses among participants of September 2009 bicycle ride, Oregon, USA*

Survey response	No. respondents/total no. participants (%)†		Ratio (95% CI)
	Internet-based survey	Telephone survey	
Initiation of survey	153/201 (76)	76/91 (84)	0.9 (0.8–1.02)
Confirmed ride participation	137/153 (90)	72/76 (95)	0.9 (0.9–1.02)
Completed survey	129/137 (94)	72/72 (100)	0.9 (0.9–0.98)
Overall completion rate	129/201 (64)	72/91 (79)	0.8 (0.9–0.98)
Completed surveys within 2 days after survey release	92/129 (71)	47/72 (65)	1.1 (0.9–1.3)
Answered 90% of questions about food items	74/129 (57)	68/72 (94)	0.6 (0.5–0.7)
Attack rates‡	23/126 (18)	13/72 (18)	1.0 (0.5–1.9)

*CI, confidence interval. †Number of riders who answered the question compared with number of riders who were asked. ‡Three respondents to the Internet-based survey who reported illness that did not meet the case definition were excluded from analysis for attack rates.

**Table 2:** Overall survey completion rate among participants in September 2009 bicycle ride, Oregon, USA, 2009

Stratification variable	No. respondents/total no. participants (%)*		p value
	Internet-based survey	Telephone survey	
Sex			
M	95/153 (62)	44/56 (79)	0.03
F	34/48 (71)	28/35 (80)	0.34
Age, y†			
<50	48/86 (56)	31/37 (84)	0.001
>50	80/114 (70)	41/54 (76)	0.44
Living accommodations			
Event organizer’s tents	36/56 (64)	18/21 (86)	0.07
Not in event organizer’s tents	93/145 (64)	54/70 (77)	0.03

*Number of riders who answered the question compared with number of riders who were asked. †Age was missing for 1 participant in the Internet-based survey.

**Figure 1:**
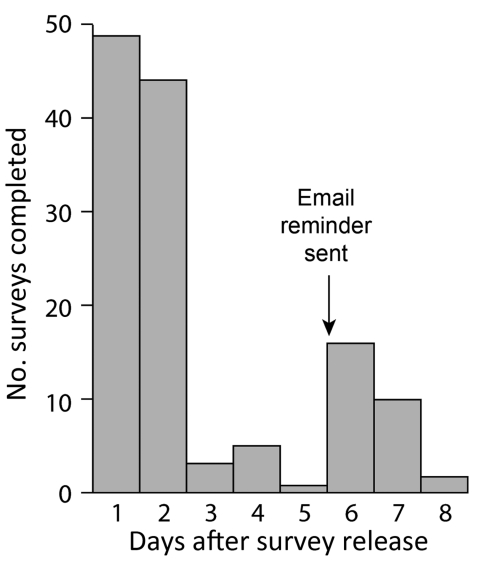
Timeliness of completed Internet-based surveys among participants in September 2009 bicycle ride, Oregon, USA.

Both cohorts completed the survey within 2 days (92/129 [71%] Internet vs. 47/72 [65%] telephone; p = 0.44) (Table 1, Figure 1). Only 74 (57%) of 129 riders who completed the Internet survey answered >90% of the food item questions, compared with 68 (94%) of 72 riders in the telephone survey (p<0.0001).

Three Internet survey respondents reported illness that did not meet the case definition; they were excluded from analysis. Among the remaining 126 Internet respondents, illness of 23 (18%) met the case definition, as did illness of 13 (18%) of 72 telephone interviewees. The attack rate for the Internet survey cohort who responded within 2 days after survey release (21/91 [23%]) was higher than for those who responded later (2/35 [6%]; p = 0.02); among telephone interviewees, percentage of cases among early interviewees (8/47 [17%] did not differ significantly from those among later interviewees (5/25 [20%]).

**Figure 2:**
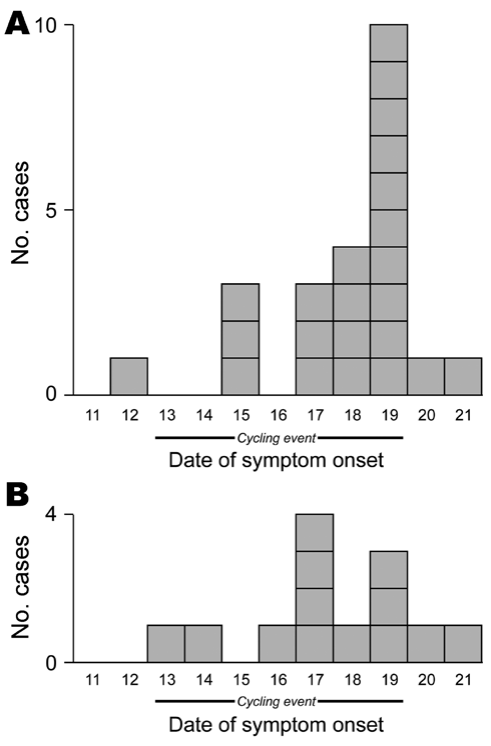
Comparison of epidemic curves in Internet-based survey (n = 23) (A) and telephone-based survey (n = 13) (B) for ill participants in September 2009 bicycle ride, Oregon, USA.

The epidemic curve appeared consistent with propagated transmission that peaked near the end of the event (Figure 2). Illness was not significantly associated with age, sex, hand-hygiene practices, reported availability of soap and water, or any of the food items in either survey cohort.

Camping in the organizer’s tents during the event was not significantly associated with illness in the telephone survey (4/18 [22%] in the organizer’s tents vs. 9/54 [17%] in other accommodations; risk ratio [RR] 1.3, 95% confidence interval [CI] 0.5–3.8). However, it was significantly associated with illness in the Internet survey (12/34 [35%] vs. 11/92 [12%]; RR 3.0, 95% CI 1.4–6.0) and in the combined dataset (Mantel-Haenszel summary RR 2.3, 95% CI 1.3–4.0).

## Conclusions

The Internet and telephone survey methods yielded similar findings with noteworthy differences. Our Internet survey response rate was comparable with that in some reports (*1*) and higher than in others (*2*,*7*). Overall, we found a lower response rate for the Internet survey cohort, with significantly fewer complete surveys. Riders ≥50 years of age were somewhat more likely to complete the Internet survey than were their younger peers in this relatively affluent cohort.

Illness was associated with use of the event organizer’s tents in the Internet survey only. Similar proportions of respondents reported illness and reported sleeping in the tents in both survey cohorts, making response bias an unlikely explanation for the different findings. Tents were reallocated at each stop; thus, riders did not use the same tent every night. Smaller sample size, leading to insufficient power in the telephone survey, could have contributed to the differing results, which might have led to different conclusions on the association of the event organizer’s tents with illness. Nonetheless, an environmental source of exposure from contaminated tents is biologically plausible, given the low infectious dose of norovirus and its ability to persist on surfaces (*8*).

Our experience is relevant to other public health agencies considering Internet surveys for outbreak investigations. First, early respondents to the Internet survey were more likely to report illness than were later respondents, suggesting that a response bias was present soon after survey release that disappeared with time and the reminder email. Survey invitations and reminders must explicitly encourage all invitees, not just those in whom illness developed, to complete the survey. Second, 1 reminder after 5 days boosted response to the Internet survey; more frequent reminders initiated earlier would have required minimal time and might have boosted overall response further. Third, a disadvantage of Internet questionnaires is the absence of a prompter to encourage survey completion and address questions. Implementing mandatory data-entry checks to advance through the survey might lead to more complete survey data. Internet survey methods might be more practically suited for relatively shorter, straightforward questionnaires that do not risk respondent fatigue and early termination and do not attempt to assess complex arrays of potential exposures that might require interviewer clarification and assistance.

This study has certain limitations. Our findings may not be generalizable to groups with different patterns of Internet access or use (*9*). Also, delays in administering our survey (the first notification came 5 days after the event) might have influenced response rates and exposure recall. Finally, we did not formally quantify and compare the costs of designing and conducting these 2 surveys.

Internet surveys will likely be increasingly used to investigate outbreaks. Our experience suggests that developing quality Internet surveys requires more initial time and effort (greater fixed cost), but once the survey instrument is deployed, it requires less time and expense per respondent for public health agencies (less variable cost). Accordingly, Internet surveys probably become more economical as the group to be surveyed becomes larger. Continued evaluations of Internet surveys are warranted to validate their findings, particularly among populations with lower Internet access and use.
